# Spinal Subarachnoid Hematoma After Cardiac Angiography in an Infant: A Case Report

**DOI:** 10.7759/cureus.38753

**Published:** 2023-05-09

**Authors:** Tsuyoshi Aihara, Itaru Hayakawa, Kenichi Usami, Hideki Ogiwara, Yuichi Abe

**Affiliations:** 1 Neurology, National Center for Child Health and Development, Tokyo, JPN; 2 Neurological Surgery, National Center for Child Health and Development, Tokyo, JPN

**Keywords:** pediatric neurosurgery, cardiac angiography, laminotomy, spinal shock, spinal subarachnoid hemorrhage

## Abstract

Spinal subarachnoid hematoma may result in sequelae such as bilateral lower extremity paralysis and vesicorectal disturbances. Although spinal subarachnoid hematoma is rare in infants, early intervention has been suggested to improve neurological prognosis. Therefore, clinicians are encouraged to make early diagnosis and surgical intervention.

A 22-month-old boy was prescribed aspirin for a congenital heart disease. A routine cardiac angiography was performed under general anesthesia. Fever and oliguria developed on the next day, followed by flaccid paralysis of the lower limbs four days later. Five days later, he was diagnosed with spinal subarachnoid hematoma and associated spinal cord shock. Even after emergent posterior spinal decompression, hematoma removal, and rehabilitation, the patient remained with bladder rectal disturbance and flaccid paralysis of both lower limbs.

Diagnosis and treatment of this case were delayed mainly because of his difficulty to complain of back pain and paralysis. The neurogenic bladder was one of the first neurological symptoms in our case, so it may be important to consider spinal cord involvement in infants with bladder compromise.

Risk factors for spinal subarachnoid hematoma in infants are largely unknown. The patient had undergone a cardiac angiography the day before the onset of the symptoms, which may be related to subarachnoid hematoma. However, similar reports are scarce, with only one case of spinal subarachnoid hematoma reported in an adult following cardiac catheter ablation. Accumulation of evidence regarding risk factors for subarachnoid hematoma in infants is warranted.

## Introduction

Spinal subarachnoid hematoma may sometimes form a hematoma compressing the spinal cord when the bleeding is rapid or accompanied by cerebrospinal fluid stasis [1]. Spinal subarachnoid hematoma may result in devastating sequelae such as paralysis of both lower extremities and cysto-rectal disturbances [2]. Because early intervention improves neurological prognosis, early diagnosis and immediate surgical intervention are important [2]. On the other hand, spinal subarachnoid hematoma in infants is very rare. In this report, we describe what is to the best of our knowledge the first case report of an infant who developed a spinal subarachnoid hematoma after cardiac angiography.

## Case presentation

The patient was a 22-month-old boy with a history of pulmonary atresia and ventricular septal defect, as well as a developmental delay due to 22q11.2 deficiency. He was able to walk independently and follow simple instructions, but he had not yet spoken his first few words. He underwent a Blalock-Taussig shunt procedure after birth and was subsequently treated with aspirin at 5 mg/kg/day. Cardiac angiography was performed on day X, which was the day we refer to when describing the subsequent events. The examination was performed under general anesthesia and there were no intraoperative complications. The patient was discharged on day X+1 as scheduled.

**Figure 1:**
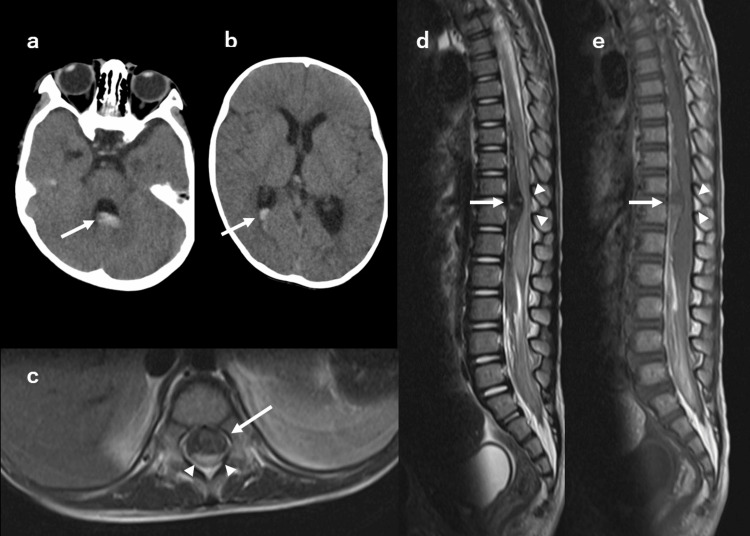
Head CT and spinal MRI. (a-b) Axial head CT without contrast at X+3 day. Acute hemorrhage is seen in the third, right lateral, and fourth ventricles (arrows). (c-e) Spinal MRI at X+5 day. (c) Axial T2-weighted MRI shows the spinal cord (arrowheads) was compressed from the ventral subarachnoid hematoma (arrow). (d-e) Sagittal images show the spinal cord (arrowhead) and the large subarachnoid hematoma (arrows) at the level of the 11th and 12th thoracic vertebrae (d: T2-weighted image, e: T1- weighted image).

In the evening of the same day, a fever of approximately 39°C, oliguria, and edema of both lower extremities appeared, and the patient visited the emergency department. At that time, there was no apparent weakness in the lower limbs. The cardiac evaluation revealed no abnormality. He was admitted to the cardiology department and followed up with a tentative diagnosis of urinary tract infection. On X+3 day, cervical rigidity appeared. Head computed tomography (CT) showed blood accumulation in the third, right lateral, and fourth ventricles (Figures 1a, 1b). There was no abnormality in the brain parenchyma. On X+4 day, flaccid paralysis of the lower limbs appeared. On X+5 day, neurological examination revealed the disappearance of both patellar tendon reflexes, urinary retention, anal sphincter muscle relaxation, and loss of the testicular elevator reflex. Spinal magnetic resonance imaging (MRI) revealed a subarachnoid hematoma at the level of the 11th and 12th thoracic vertebrae, which compressed the spinal cord from the ventral side (Figures 1c-1e). No vascular malformations in the spinal cord were identified on contrast-enhanced MRI, and spinal angiography was not performed. Blood coagulation function was not evaluated immediately before the catheter examination, but there were no abnormalities in subsequent evaluations after onset. No other findings suggestive of coagulation abnormalities were observed since birth. We did not find any obvious history of trauma, but we could not completely rule out the possibility of minor injuries.

**Figure 2:**
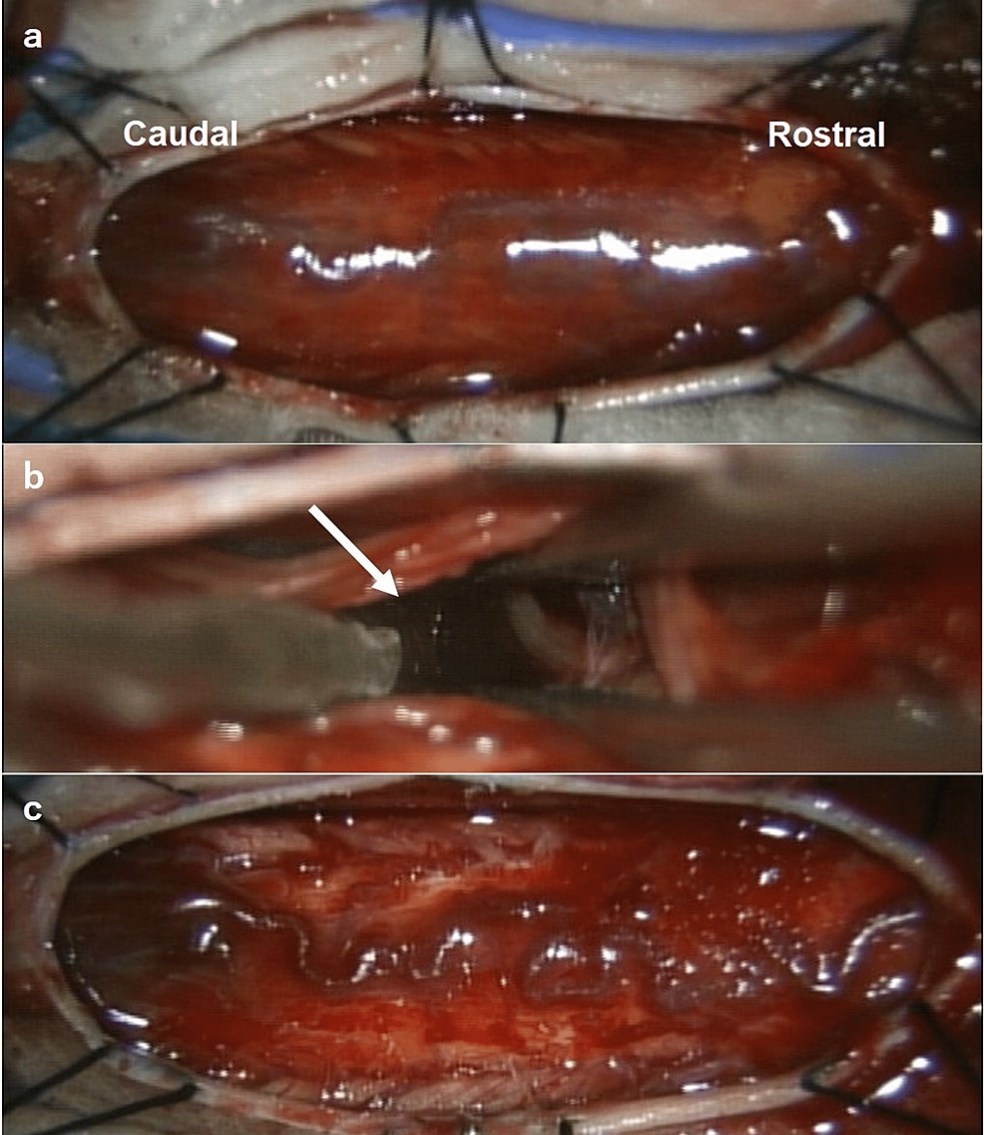
The laminotomy procedure. (a) After laminotomy and dural opening of the 11th and 12th thoracic vertebrae, the spinal cord was protruding dorsally due to ventral compression by the hematoma. (b) The hematoma (arrow) was removed avoiding the spinal cord. (c) After hematoma removal, the spinal cord overhang returned to normal.

The diagnosis of spinal cord shock due to subarachnoid hematoma (ventral type) was made. About 48 hours after the onset of the flaccid paralysis, posterior decompression was performed by laminotomy of the 11th and 12th thoracic vertebrae and evacuation of the hematoma. The surgeon confirmed that the hematoma was located in the subarachnoid space. After hematoma removal, the spinal cord was deviated ventrally (Figure 2). Despite aggressive rehabilitation, three months later, the patient remained with bladder rectal disturbance requiring urinary drainage and enema and flaccid paralysis of both lower extremities (manual muscle testing was 2).

## Discussion

We report an infant who developed a hematoma due to spinal subarachnoid hematoma after cardiac angiography and presented with paralysis of both lower extremities and cysto-rectal disturbances. Spinal subarachnoid hematoma is rare, occurring in 1% of intracranial subarachnoid hematoma [3]; however, when it does occur, it may result in severe sequelae [2].

The patient was left with paralysis of both lower extremities and bladder and rectal disturbances as sequelae. Patients with spinal cord symptoms for more than 24 hours after onset have a poor prognosis. Spinal decompression and hematoma removal should be performed without delay [4,5]. In adults, complaints of back pain often lead to a diagnosis [2]. However, the diagnosis may be delayed in infants because of their difficulty in expressing symptoms [6]. In the present case, pain could not be captured throughout the entire course of the disease. Furthermore, the lower extremity tendon hyporeflexes became evident on X+5 day, while oliguria due to neurogenic bladder developed on X+1 day. Thus, it is important to consider a spinal lesion, including spinal subarachnoid hematoma, as a differential cause of acute urinary retention in infants and toddlers. Timely imaging of the spine is essential to reduce neurological sequelae.

The use of antiplatelet agents and endovascular manipulation may have caused the bleeding in our case. A number of causes of spinal subarachnoid hematoma have been reported in the literature, including vascular malformations, anticoagulant status, epidural anesthesia, systemic lupus erythematosus, Behcet’s disease, Marfan syndrome, schwannoma, and idiopathic spinal subarachnoid hematoma with an unknown cause [2,7-10]. In a systematic review of 95 cases of spinal subarachnoid hematoma, only one case involved an infant under five years of age [2]. Others reported complications of spinal subarachnoid hematoma in pediatric-onset conditions include hemophilia A [11], Klippel-Trenaunay-Weber syndrome [12], spinal meningioma [13], and neurofibromatosis type 2 [14]. In this case, two possible risks for hematoma were that the patient was being administered antiplatelet medications and had undergone a cardiac angiography the day before the onset of the hemorrhage. Although there has been a case report of spinal subarachnoid hemorrhage after catheter ablation in an adult [15], to our knowledge, this is the first report of spinal subarachnoid hematoma after angiography in infants. Although the mechanism leading to the hemorrhage is unknown, we speculated that ischemia and reperfusion of the Adamkiewicz artery following catheterization may have caused the spinal subarachnoid hematoma. Based on this case, it is important to recognize that angiography may also be a risk factor for spinal subarachnoid hematoma, although this is a rare complication.

Spinal subarachnoid hematoma can also cause delayed cerebral infarction due to cerebral vasospasm [16]. This is caused by an influx of blood into the cranium after a spinal subarachnoid hematoma. Blood concentrates around the arachnoid granulation, which absorbs cerebrospinal fluid, and can be observed on CT [16]. In the present case, CT of the head on X+3 days showed hematoma in the dorsal horn of the lateral, third, and fourth ventricles; however, fortunately, no secondary cerebral infarction was observed. Patients with spinal subarachnoid hematoma should be carefully monitored for the appearance of new abnormal neurological findings suspicious of cerebral infarction, especially around one week after the spinal subarachnoid hematoma.

## Conclusions

Spinal subarachnoid hematoma should be considered as a differential diagnosis in cases of cysto-rectal disturbances in infants, especially those at risk for bleeding. Although the mechanism is unknown, the use of antiplatelet agents and cardiac angiography may be risk factors for spinal subarachnoid hematoma that warrant further investigation.

## References

[1] Komiyama M, Yasui T, Sumimoto T, Fu Y (1997). Spontaneous spinal subarachnoid hematoma of unknown pathogenesis: case reports. Neurosurgery.

[2] Kreppel D, Antoniadis G, Seeling W (2003). Spinal hematoma: a literature survey with meta-analysis of 613 patients. Neurosurg Rev.

[3] Walton JN (1953). Subarachnoid haemorrhage of unusual aetiology. Neurology.

[4] Pau A, Brambilla M, Cossu M, Francaviglia N, Siccardi D, Silvestro C (1991). Spinal subarachnoid hematoma of unknown etiology. A case report. Neurochirurgia (Stuttg).

[5] Plotkin R, Ronthal M, Froman C (1966). Spontaneous spinal subarachnoid haemorrhage. Report of 3 cases. J Neurosurg.

[6] Takahashi Y, Hayakawa I, Abe Y (2021). Diagnostic odyssey of acute disseminated encephalomyelitis in children. Sci Rep.

[7] Domenicucci M, Ramieri A, Paolini S, Russo N, Occhiogrosso G, Di Biasi C, Delfini R (2005). Spinal subarachnoid hematomas: our experience and literature review. Acta Neurochir (Wien).

[8] Fody EP, Netsky MG, Mrak RE (1980). Subarachnoid spinal hemorrhage in a case of systemic lupus erythematosus. Arch Neurol.

[9] Wityk RJ, Zanferrari C, Oppenheimer S (2002). Neurovascular complications of marfan syndrome: a retrospective, hospital-based study. Stroke.

[10] Zhang HM, Zhang YX, Zhang Q, Song SJ, Liu ZR (2016). Subarachnoid hemorrhage due to spinal cord schwannoma presenting findings mimicking meningitis. J Stroke Cerebrovasc Dis.

[11] Choudhary AK, Jha B (2011). Imaging findings in spinal subarachnoid hemorrhage in patient with hemophilia A: an unusual cause for back pain. Emerg Radiol.

[12] Komatsu Y, Kuzuhara S, Kanazawa I, Nakanishi T (1985). [Klippel-Trénaunay-Weber syndrome associated with spinal arteriovenous malformation--a case report]. Rinsho Shinkeigaku.

[13] Yusefovic T, Avram J, Tiberin P (1978). Long-standing myelomeningocele associated with spinal subarachnoid hemorrhage. Case report. Childs Brain.

[14] Inoue T, Miyamoto K, Kushima Y, Kodama H, Nishibori H, Hosoe H, Shimizu K (2003). Spinal subarachnoid hematoma compressing the conus medullaris and associated with neurofibromatosis type 2. Spinal Cord.

[15] Cooley-Rieders K, Paredes K (2020). Rare pathology leading to a diagnostic challenge: a subarachnoid spinal hematoma after catheter cryoablation for atrial fibrillation. J Cardiol Cases.

[16] Shakur SF, Farhat HI (2013). Cerebral vasospasm with ischemia following a spontaneous spinal subarachnoid hemorrhage. Case Rep Med.

